# Perinatal Caffeine, Acting on Maternal Adenosine A_1_ Receptors, Causes Long-Lasting Behavioral Changes in Mouse Offspring

**DOI:** 10.1371/journal.pone.0003977

**Published:** 2008-12-18

**Authors:** Olga Björklund, Johan Kahlström, Peter Salmi, Bertil B. Fredholm

**Affiliations:** Department of Physiology and Pharmacology, Karolinska Institutet, Stockholm, Sweden; Lund University, Sweden

## Abstract

**Background:**

There are lingering concerns about caffeine consumption during pregnancy or the early postnatal period, partly because there may be long-lasting behavioral changes after caffeine exposure early in life.

**Methodology/Principal Findings:**

We show that pregnant wild type (WT) mice given modest doses of caffeine (0.3 g/l in drinking water) gave birth to offspring that as adults exhibited increased locomotor activity in an open field. The offspring also responded to cocaine challenge with greater locomotor activity than mice not perinatally exposed to caffeine. We performed the same behavioral experiments on mice heterozygous for adenosine A_1_ receptor gene (A_1_RHz). In these mice signaling via adenosine A_1_ receptors is reduced to about the same degree as after modest consumption of caffeine. A_1_RHz mice had a behavioral profile similar to WT mice perinatally exposed to caffeine. Furthermore, it appeared that the mother's genotype, not offspring's, was critical for behavioral changes in adult offspring. Thus, if the mother partially lacked A_1_ receptors the offspring displayed more hyperactivity and responded more strongly to cocaine stimulation as adults than did mice of a WT mother, regardless of their genotype. This indicates that long-term behavioral alterations in the offspring result from the maternal effect of caffeine, and not a direct effect on fetus. WT offspring from WT mother but having a A_1_R Hz grandmother preserved higher locomotor response to cocaine.

**Conclusions/Significance:**

We suggest that perinatal caffeine, by acting on adenosine A_1_ receptors in the mother, causes long-lasting behavioral changes in the offspring that even manifest themselves in the second generation.

## Introduction

Caffeine (1,3,7-trimethylxanthine) is a widely consumed psychoactive substance that is readily available through several dietary products (coffee, tea, cocoa beverages and chocolate bars). The total worldwide consumption of caffeine (irrespective of source) has been estimated to approximately 70 to 76 mg/person/day. Interestingly, the levels of caffeine intake in countries such as Sweden and Finland reach more than 400 mg/person/day [1]. Although health consequences of ordinary caffeine consumption are probably minor there are concerns about caffeine intake during pregnancy and lactation. It is notable that in contrast to alcohol and tobacco consumption during pregnancy, approximately 70% of expectant mothers continue to drink beverages containing caffeine at normal or near normal rate [2], [3].

Human and animal studies have shown that high caffeine intake represents a risk for adverse pregnancy outcomes and teratological consequences in offspring [4], [5]. There are many animal studies on the effect of caffeine intake by dams, and often rather high doses of this substance (>50 mg/kg) have been studied. It is therefore important to note that the behavioral effects of caffeine are characterized by a biphasic dose-effect relationship. At low to moderate doses (50 to 300 mg, i.e. 1 to 3 cups of coffee), caffeine induces a central stimulation in humans, eliciting feelings of wellbeing, alertness, energy and ability to concentrate. In contrast, the subjective effects induced by caffeine at higher doses (300 to 800 mg) are characterized by negative feelings such as anxiety, nervousness and insomnia, a condition sometimes referred to as “caffeinism” [6]. In laboratory animals the behavioural effects of caffeine are also biphasic [7]. For example, low doses (<25 mg/kg) of caffeine are similar to psychomotor stimulants such as cocaine and amphetamine, whereas at higher doses caffeine has effects that are similar to a diverse set of other agents such as benzodiazepine-inverse agonists and phencyclidine (PCP) [8].

In low doses, which are the most relevant to human use, caffeine effects are exerted by antagonizing brain adenosine A_1_ and A_2A_ receptors with secondary effects on dopaminergic neurotransmission [1]. There is little evidence that these doses produce teratological effects [9]. One concern about early exposure to low or modest doses of caffeine relates to hyperactivity in late adolescence or adulthood [10]. Early exposure to psychostimulant drugs may lead to a phenomenon called “neuronal imprinting” where a drug may have effects that are not necessarily immediate but manifest later in life [11]. Relating to the fact that the rewarding properties of all psychostimulants, including to some extent caffeine [7], are a result of actions of the drugs on the mesolimbic dopamine system [12], early exposure to caffeine might also produce late consequences e.g. in the reaction to other psychoactive drugs.

It is often tacitly assumed that the reason that psychoactive drugs have long-term behavioral consequences is due to the affected fetal brain. However, it is clearly also possible that the drug affects maternal physiology or behavior in such a way that there are long-term consequences in the offspring. It will always be difficult to discriminate between these two possibilities when only drug administration is used. We therefore wanted to see if aspects of the effects of caffeine could be mimicked by genetic targeting of one of the adenosine receptors, since this might allow a separation into maternal or filial effects.

The present study was designed to further assess the behavioural status, including motor functions and psychomotor activation of adult animals whose mothers were exposed to caffeine during pregnancy and lactation and their response to the psychostimulant cocaine. The rationale behind testing the response to another psychoactive stimulus in these animals is that caffeine, itself a motor stimulant, might be expected to change the motor activity in response to cocaine. Some neurochemical measurements were also made. We found that some effects of perinatal exposure to caffeine were mimicked in mice heterozygous for adenosine A_1_ receptors, which have half the normal number of such receptors. This is relevant because regular human consumption of caffeine leads to the blockade of about half of the body's A_1_ receptors.

## Results

### Perinatal caffeine exposure

**Figure 1:**
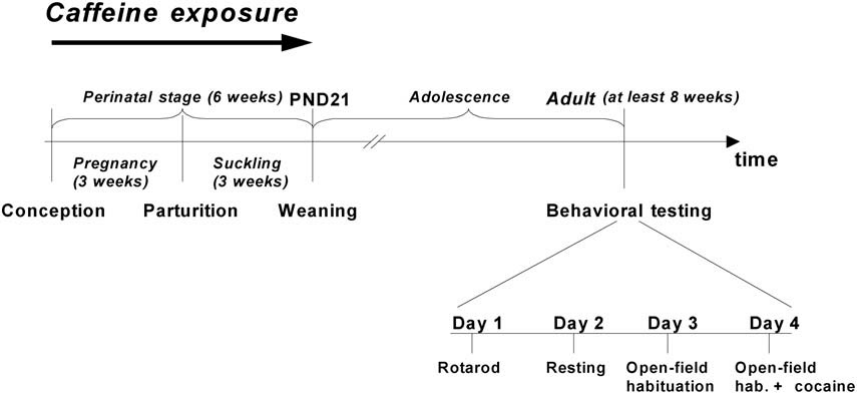
Experimental design. Pregnant WT females were divided in two groups: one exposed perinatally to 0.3 g/l caffeine (WT Caff), the other receiving tap water (WT H_2_O) from GD1 to day 21 of lactation (PND21). After the separation from the mother, the offspring were housed 2–8 per cage (males and females separately). At 2 months of age mice were tested at the rotarod, and then allowed to rest for one day and on the day 3 let to spontaneously explore an open field for two separate 45 min sessions. The sessions were separated by a 2 h resting period. Following the open-field habituation and test on day 4, the animals were sacrificed by means of decapitation and their brains were dissected out and stored at −80°C.

Adult WT mice, 8–10 weeks of age, were perinatally exposed to 0.3 g/l caffeine in the drinking water given to the dams from GD1 to PND21. The dose of caffeine given produced blood levels in dams comparable to those obtained in humans after consumption of 3–4 cups of coffee [13]. Together with offspring of untreated WT dams, caffeine pre-treated male and female mice were tested for motor balance and coordination on the rotarod on Day 1 of the evaluation protocol (Figure 1). There were no major differences in either sex between untreated group and mice given perinatal caffeine in terms of their ability to remain on the rotarod (Figure S1. A,B of the supplemental data).

**Figure 2:**
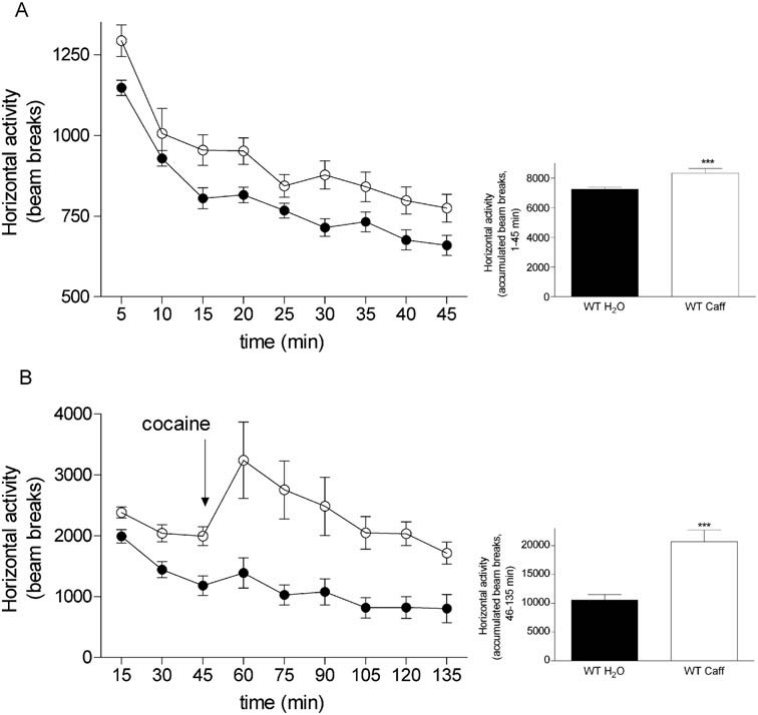
Spontaneous horizontal activity measurements after caffeine exposure. (A) Spontaneous horizontal activity measurements in adult wild type females exposed to 0.3 g/l caffeine (WT Caff) from GD1 to PND21 (white circle) and WT group receiving tap water (WT H_2_O, black circle). Each point represents the mean±S.E.M of the beam breaks recorded during 5 minute intervals. Statistical analysis was performed with Student's t test (females WT H_2_O, n = 25, WT Caff n = 12, ***p<0.01). (B) Analysis of open field behavior in two month-old female mice after stimulation with cocaine (10 mg/kg, 90 min recording). Horizontal activity was recorded during habituation 3 (min 1–45) and after cocaine stimulation (min 46–135). Each point represents the mean±S.E.M (females WT H_2_O n = 8, WT Caff n = 7) of the beam breaks recorded during 15 minute intervals for habituation 3 and cocaine challenge. Arrows indicate the time of cocaine injection. Statistical analysis was performed using Student's t test (***p<0.001 significantly different from WT H_2_O).

Two periods of habituation to the open field were preformed on Day 3 and the spontaneous activity was evaluated in all groups tested. As shown in Figure 2A caffeine pre-treated adult WT females displayed increased (121.7±2.1) spontaneous motor activity compared to the WT untreated females (113.3±1.2) (p = 0.0008, t = 3.68, df = 35 Student's t test). Adult WT males pre-treated with caffeine also had significantly increased horizontal activity during the 45 min of testing compared to the WT untreated group as already published [14]. In agreement with these results, male and female offspring from our other recent study, where the wild type mothers were treated with the same concentration of caffeine (0.3 g/l) but from GD7 to PND7, also displayed higher locomotor motor activity as adults [14]. This implies that even a narrower time window of caffeine exposure during development might be sufficient in inducing life-long consequences.

The WT male mice perinatally exposed to caffeine displayed a more pronounced increase in locomotor activity than controls after the cocaine injection [14]. Caffeine pre-treated female mice were also characterized by a higher response to cocaine stimulation than the controls during the 90 min recording session (Figure 2B) (Student's t test for Ha: WT H_2_O 46.5±3.5, n = 8; WT Caff 73.8±4.7, n = 7, p = 0.0004, t = 4.75, df = 13). This observation was similar to our previous report on the higher effect of amphetamine stimulation in caffeine pre-treated female offspring [14].

### Response to cocaine stimulation in A_1_R KO and A_2A_R KO mice

**Figure 3:**
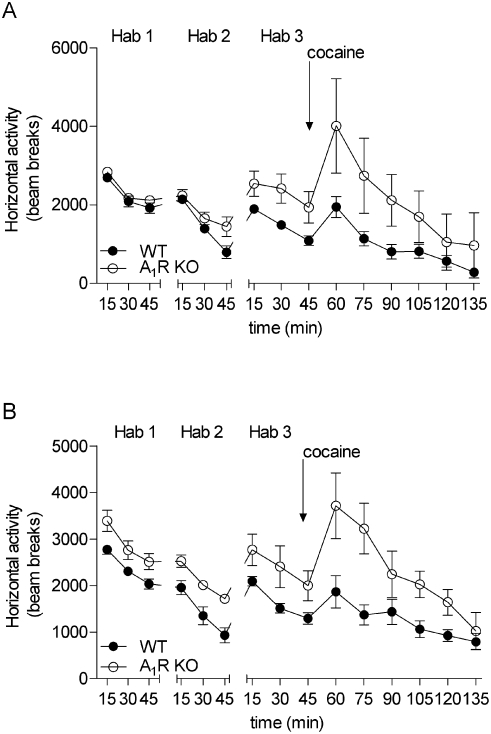
Open field behavior of A_1_R KO mice. During habituation 1, 2 on the first day of examination and habituation 3 (45 min), followed by 10 mg/kg cocaine injection (90 min) in WT (black circle) and A_1_R KO (open circle) male (A) and female (B) mice, in the second day of testing. Arrows indicates the time of cocaine injection. Each point represents the mean±S.E.M of the beam breaks recorded during 15 minute intervals (males WT n = 10, A_1_R KO n = 3; females WT n = 12, A_1_R KO n = 4).

It is known that behavioural effects of 15 mg/kg caffeine can be largely accounted for by blockade of adenosine receptors and that A_1_ and A_2A_ receptors are particularly important [1]. We examined if complete elimination of A_1_R would mimic the effect of caffeine. As seen in Figure 3A,B cocaine injection (10 mg/kg) induced a higher locomotor activity in the A_1_R KO adult male mice than in the wild types (accumulated beam breaks for 90 min following injection for Ha, WT: 39.5±2.6, n = 10, A_1_R KO: 63.9±14.4, n = 3, p = 0.008, df = 11 Student's *t* test). This was partially due to a higher basal activity (Figure 3A). The difference in cocaine response was, if anything, more pronounced in female mice from A_1_R KO genotype than in the WT group of the same sex (Ha, accumulated beam breaks from 46–135 min after cocaine injection: WT 51.6±3.7, n = 12, A_1_R KO: 71.6±6.9, n = 4, p = 0.009, df = 14 Student's *t* test), in part as a consequence of a higher basal activity (Figure 3B).

Whereas A_1_R KO mice appear to be more responsive to cocaine than wild type animals the opposite is known to be true for A_2A_R KO mice since several previous reports, from our and other groups, have demonstrated decreased response to cocaine or amphetamine in animals that lack A_2A_ receptor gene [14], [15].

The obtained results, higher response of A_1_R KO animals, as well as impaired response in A_2A_R KO mice to cocaine stimulus reported in the literature, prompted us to continue the work involving adenosine A_1_ receptor. Since normal doses of caffeine block half the A_1_ receptors in the body [16], we used mice heterozygous for adenosine A_1_ receptors as comparison to the WT perinataly caffeine treated mice.

### A_1_R Hz mice

**Figure 4:**
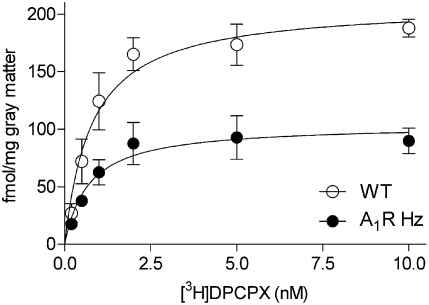
[^3^H]DPCPX-binding in the Nucleus accumbens. Saturation curve of [^3^H]DPCPX-binding in the Nucleus accumbens from WT and A_1_R Hz mice. Significant difference in B_max_ values for the two groups was found by Student's t test (B_max_ WT 208±8.23 fmol/gray matter, n = 6, A_1_R Hz 104±5.36 fmol/gray matter, n = 6, df = 10, ***p<0.001). The same was observed in striatum.

Autoradiography with the selective A_1_R antagonist [3H]DPCPX determined that half of the number of A_1_R present in A_1_R Hz mice than in the WT when the area of nucleus accumbens was examined (Figure 4) and striatum (not shown).

When A_1_R Hz animals were tested for motor coordination on the rotarod, decreased fall latency was observed in A_1_R Hz males, but no changes in the ability to remain on the rotating rod were found in A_1_R Hz females compared to the wild types (Figure S1. A,B of the supplemental data, males: WT H_2_O 124.2±7.4, n = 21 and A_1_R Hz 94.0±9.8, n = 8, p = 0.03, t = 2.2, df = 27 Student's t test).

**Figure 5:**
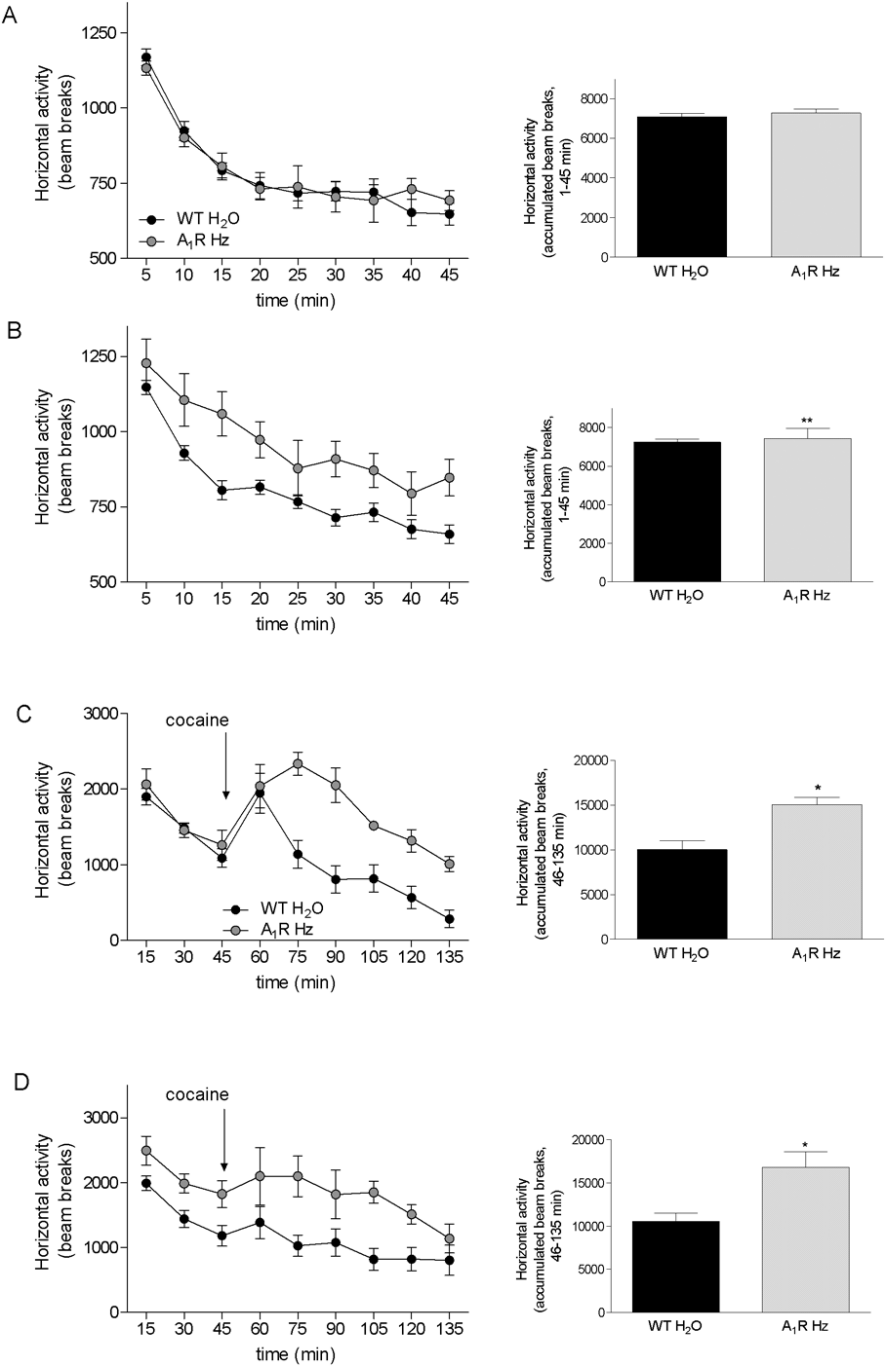
Spontaneous horizontal activity of A_1_R Hz mice. Measurements of spontaneous horizontal activity in adult male (A) and female (B) wild type and A_1_R Hz mice. WT group receiving tap water (WT H_2_O: males n = 17, females n = 25, black circle) and A_1_R Hz group (males n = 7, females = 8, gray circle). Each point represents the mean±S.E.M of the beam breaks recorded during 5 minute intervals. Statistical analysis was performed with Student's t test (**p<0.01). (C) and (D) Analysis of open field behavior after stimulation with cocaine in adult mice (10 mg/kg, 90 min recording). Black circle: offspring from control (WT H_2_O: males n = 11, females n = 8) dams, gray circle: A_1_R Hz offspring (males n = 3, females n = 4). Horizontal activity was recorded during habituation 3 (min 1–45) and after cocaine stimulation (min 46–135). Each point represents the mean±S.E.M of the beam breaks recorded during 15 minute intervals for habituation 3 and cocaine challenge. Arrows indicate the time of cocaine injection. Statistical analysis was performed with Student's t test (*p<0.05).

When tested for the spontaneous activity A_1_R Hz male group did not show a significant increase in locomotion compared to the WT untreated group (Figure 5A) (45 min of habituation 1, Ha: WT H_2_O 110.7±1.0, n = 17; A_1_R Hz 113.6±1.6, n = 7, p = 0.15, t = 1.51, df = 22, Student's t test). A statistically significant increase in locomotion was, however, displayed by female A_1_R Hz group compared to their WT controls (Figure 5B). Student's t test was run on the score of accumulated beam breaks for the 45 min period of the first habituation, WT H_2_O 113.3±1.2, n = 25 and A_1_R Hz 124.1±4.1, n = 8, p = 0.0018, t = 3.4, df = 31.

The enhanced response to cocaine challenge found in WT caffeine pre-treated male groups was also observed in the mice that were heterozygous for the A_1_ receptor gene (Figure 5C) (accumulated beam breaks for 90 min recording of Ha: WT H_2_O 42.4±3.7, n = 11; A_1_R Hz 63.3±1.1, n = 3, p = 0.02, t = 2.8, df = 12 Student's t test). A_1_R Hz female mice were also characterized by a higher response to cocaine stimulation than the controls during the 90 min recording session (Figure 5D) (Student's t test, WT H_2_O 46.5±3.5, n = 8, A_1_R Hz 63.8±4.7, n = 4, p = 0.02, df = 10).

Both adenosine A_1_ receptor heterozygous mice and WT mice exposed to perinatal caffeine showed the tendency towards increased locomotion and decreased habituation (Figure 5). Thus, the behavioral profile of A_1_R Hz mice which were born by A_1_R Hz mothers was basically similar to that of mice exposed to perinatal caffeine. However, part of the increase in response to cocaine in the mice perinatally treated with caffeine or the A_1_R Hz group could be due to a higher basal activity since those two groups tended to habituate less completely to the open field arena than the control animals (Figure 5C,D).

### Role of maternal genotype

The fact that some of the characteristics of perinatal caffeine exposure could be mimicked by partial deletion of a gene opens the possibility of examining whether it is effects in the mother or in the offspring during the perinatal period that determine the phenotype of the offspring in adulthood. We therefore compared the behavior of wild type pups and pups heterozygous for adenosine A_1_ receptors born to and raised by mothers heterozygous for adenosine A_1_ receptors, with that of pups heterozygous for adenosine A_1_ receptors born to and raised by wild type mothers. The latter was achieved by mating WT dams with A_1_R KO male mice.

**Figure 6:**
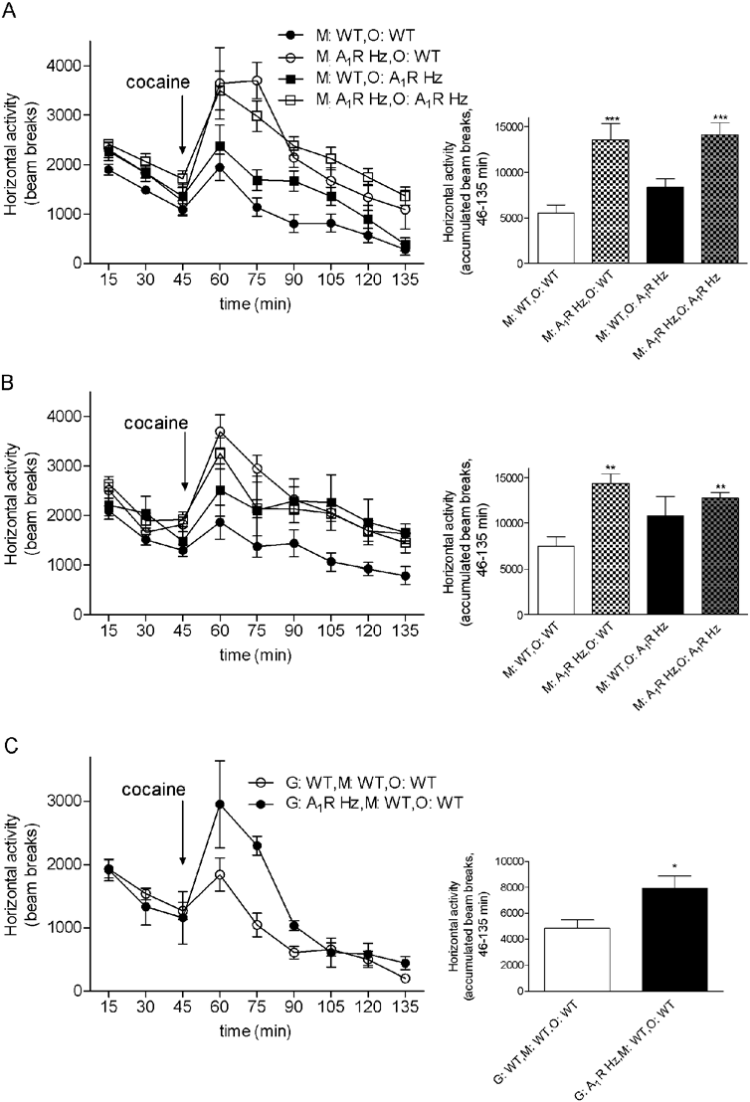
Effect of mother's genotype on cocaine responce. A_1_R Hz mice are similar to mice exposed to perinatal caffeine in locomotor response to cocaine and this response seems to be dependent on mother's genotype, not offspring's (A,B). Closed circle represents WT mice born to a WT mother (M∶WT,O∶WT, males n = 11, females n = 12), open circle WT are mice born to an A_1_R Hz mother (M∶WT,O∶A_1_R Hz, males n = 6, females n = 6), closed square stand for A_1_R Hz mice born to a WT mother (M∶A_1_R Hz,O∶WT, males n = 6, females n = 3) and open square A_1_R Hz mice born to a A_1_R Hz mother (M∶A_1_R Hz,O∶ A_1_R Hz, males n = 12, females n = 10). Mice were habituated to the open-field arena for 45 min before administration of cocaine (10 mg/kg, i.p.), immediately followed by a 90-min test session. Arrow indicates time of injection of cocaine or vehicle. Results are shown as means±S.E.M.. The enhanced response to cocaine was only present in offspring born to a mother heterozygous for the adenosine A_1_ receptor gene. Statistical analysis was performed with Two-Way ANOVA with factors offspring's genotype and factor mother's genotype (**p<0.01; ***p<0.001 significantly different from WT mother WT offspring). (C) Response to cocaine in the second generation. Open circle grandmother WT, mother WT, offspring WT (G∶WT,M∶WT,O∶WT, n = 9) and closed circle: grandmother A_1_R Hz, mother WT, offspring WT (G∶A_1_R Hz,M∶WT,O∶WT n = 4). After 45 min of habituatain mice were injected cocaine and analysed for the next 90 min. Each point represents the mean±S.E.M of the beam breaks recorded during 15 minute intervals for habituation 3 and cocaine challenge. Arrows indicate the time of cocaine injection. Statistical analysis was performed with Student's t-test (*p<0.05).

As shown in Figure 6A, there was a statistically significant increase in locomotor activity in male mice (regardless of their genotype) to the response to cocaine injection only when born to a mother that partially lacked adenosine A_1_ receptors (significant interaction being mother's genotype p<0.0001, F_(1,31)_ = 23.8, Two Way ANOVA with factors offspring and mother's genotype). The same phenomenon was also found in the female offspring of the A_1_R Hz mothers after the cocaine stimulation (interaction mother's genotype p = 0.0014, F_(1,27)_ = 12.6, Two Way ANOVA) (Figure 6B).

Furthermore, as exemplified by female mice in Figure 2B and 5B, the habituation profile of adenosine A_1_ receptor heterozygous offspring was similar to that of mice exposed to perinatal caffeine. Thus, the hyperactivity profile in offspring seems to be strongly dependent on whether the mother was heterozygous for adenosine A_1_ receptors or not. We have also preformed the statistical analysis of all our data taking into consideration the litter-related issues and observed that the significant results remained.

For additional evaluation of the effect of knocking out one copy of the mother's A_1_R gene on the second generation of the offspring we have examined the behaviour of the WT male mice whose mothers were WT but grandmothers were either WT or A_1_R Hz mice. After stimulation with cocaine we could still observe modifications caused by the absence of A_1_R on the offsprings' reaction to the psychostimulant (Figure 6C) (accumulated beam breaks from 46–135 min in Ha: WT grandmother WT mother WT offspring: 39.8±3.3, n = 9; A_1_R Hz grandmother WT mother WT offspring: 51.7±3.8, n = 4, p = 0.02, t = 2.1, df = 11 Student's t-test).

### Expression of immediate early genes and dopamine receptor subtypes

Some attempts were made to find a neurochemical correlate to the behavioral changes. The dose of cocaine used was too low to induce any significant change in the expression of either NGFI-A mRNA or c-fos in caudate-putamen, nucleus accumbens core, nucleus accumbens shell and medial prefrontal cortex in the WT mice pre-treated with caffeine and the WT controls of both sexes. Also the tyrosine hydroxylase in substantia nigra, Table 1 and [^3^H]mazindol binding in caudate-putamen (fmol/mg protein, offspring from the WT mothers: 418.8±26.3, n = 11; offspring from the A_1_R Hz mother: 362.7±21.0, n = 26, p = 0.07, t = 1.5, df = 35 Student's t-test), failed to demonstrate any significant difference. We also found no changes in the binding of dopamine D_1_ (with SCH23 390) or D_2_ (with raclopride) receptor ligands nor in expression of preprotachykinin, prodynorphin, prosomatostatin mRNA levels in nucleus accumbens or caudate-putamen (Table 1), but there was a significant increase in preproenkephalin mRNA in the offspring born to A_1_R Hz mother in the caudate-putamen (Table 1).

**Table 1:** Expression of mRNA levels in cocaine-treated offspring to adenosine A_1_R Hz or WT mother.

		Cx	NAcc	CP	SN
c-fos	WT	0.25±0.02		0.27±0.03	
	A1R Hz	0.23±0.01 ns		0.24±0.01 ns	
NGFI-A	WT	0.55±0.02	0.19±0.01	0.37±0.02	
	A1R Hz	0.54±0.01 ns	0.22±0.01 ns	0.42±0.01*	
Proenkephaline A	WT		0.75±0.03	0.68±0.02	
	A1R Hz		0.77±0.02 ns	0.75±0.01**	
Substance P	WT		0.94±0.02	0.85±0.02	
	A1R Hz		0.94±0.02 ns	0.81±0.01 ns	
Dynorphine	WT		0.35±0.03	0.26±0.01	
	A1R Hz		0.31±0.02 ns	0.24±0.01 ns	
Somatostatin	WT		0.45±0.04	0.33±0.06	
	A1R Hz		0.52±0.03 ns	0.35±0.02 ns	
Tyrosine hydroxylase	WT				0.74±0.02
	A1R Hz				0.73±0.02 ns

Cx = cortex, CP = caudatum putamen, NAcc = nucleus accumbens, SN = substantia nigra. Values are means±S.E.M of the optical density (n = 11–30). Statistical analysis was performed with the Student's *t* test (ns>0.05, ^*^p<0.05, ^***^p<0.001).

## Discussion

We have confirmed that early exposure to caffeine leads to altered motor behavior, including enhanced responses to cocaine [9], [14], [17], [18], [19]. It is recognized that low doses of cocaine and caffeine produce additive effects [20], [21]. However, in the present experiments caffeine was administered long before cocaine and another explanation than the direct drug interaction must be sought. In most, but not all of these earlier studies, perinatal caffeine in doses similar to those we used caused a slight hyperlocomotion of the offspring even in the absence of a drug challenge. Caffeine and cocaine are both behavioral stimulants, but use somewhat different mechanisms to produce their effects [22], [23]. The mechanisms underlying the behavioral features associated with caffeine, and psycho-stimulants in general, are believed to be related to the activation of dopamine (DA) receptors in the mesolimbic dopamine system (notably in the nucleus accumbens). Whereas cocaine and amphetamine primarily stimulate D_1_ receptor, caffeine largely acts by enhancing D_2_ receptor pathways [23], [24]. The effects of caffeine is believed to be indirect and due to blockade of adenosine A_2A_ receptors that are co-localized with D_2_ receptors that have opposite actions [1].

We found in our recently published study that an even shorter period of maternal caffeine intake (two weeks prenatally and one week postnatally) was sufficient to produce long-lasting behavioral changes in the offspring [14]. This suggested that the antenatal period is particularly important as also attested by the previous cross-fostering experiments [25]. We cannot rule out that also shorter periods of caffeine treatment before the pregnancy could have effects, but most data on humans suggest a complete return of physiological functions after cessation of caffeine use, for reference see [1]. In female mice, we found no evidence that long term oral caffeine intake in the doses given here alters overall activity, or the location of activity (central vs. peripheral), which may be somewhat related to anxiety following a period of caffeine exposure (Eriksson, Yang, Salmi and Fredholm, unpublished data). Furthermore, we did not observe any gross alteration in maternal behavior in the caffeine treated dams. We therefore favour the hypothesis that maternal caffeine use during a critical period of fetal development is the most important.

In practically all previous studies on perinatal caffeine effects, the focus in the discussion has been on the fetus. Indeed, caffeine readily crosses the placenta without metabolism and partially enters breast milk [26]. The human fetus and newborn infant is exposed to caffeine for a prolonged period of its early life as liver enzymes which metabolize caffeine are not present until eight months of age [26]. However, it is clearly also possible that the relevant action is on the mother. Previous cross-fostering experiments have demonstrated that the effect of a single caffeine dose on the subsequent behavior could not be ascribed to changes in mother's behavior or lactational efficiency in mice [25]. It could, however, be due to an altered uterine environment [27], [28], [29]. It has been shown that a single, very high dose of caffeine (120 mg/kg) could reduce blood flow to the uterus and decidua [30]. This may cause changes in fetal oxygen supply and/or changes in maternal blood composition, but the effects of such high doses are very different from what is observed with the present dosing. However, to completely differentiate between maternal and fetal actions in this period will be virtually impossible when examining drug administration, which by necessity must be through the mother. This is why we tried to find a genetic model that could mimic some aspects of exposure to caffeine.

The present data show that several features of perinatal caffeine administration, notably the enhanced response to cocaine, can be mimicked by deletions of the A_1_ receptor, a known target of caffeine, whereas it was known since before that deletion of both copies of the A_2A_ receptor had the opposite effect [15]. We also found that the deletion of only one of the copies of the A_1_ receptor gene, resulting in approximately half the number of receptors in cortex and hippocampus [16] but also in regions of more direct relevance here (present data), enhanced the response to cocaine. By contrast, deletion of one of the copies of the A_2A_ gene reduces the response to cocaine (Jiang-Fan Chen, personal communication) albeit not to the same extent as removal of both copies [15]. It is important to note that the A_1_ heterozygous mouse still responds to adenosine, but approximately twice as much of the agonist is needed for the same response [16], [31]. Such a parallel shift of the dose-response is also achieved by caffeine at concentrations close to the K_d_ for the antagonist (10–30 µM), concentrations that are attained by the doses of caffeine used in the present experiments.

It is also noteworthy that the effect of caffeine on the behavior of the offspring was (at least partially) mirrored by the A_1_ receptor deletion, whereas the stimulatory responses of caffeine are generally dependent on the A_2A_ receptor [32], [33] although A_1_ receptors contribute [34], [35], [36], [37]. This suggests that the effect is not simply due to psychostimulant actions where A_2A_ receptors are very important, but that some other features must be involved. It is probably relevant that we have previously demonstrated that the ability of a low dose of caffeine to cause reinstatement of cocaine-seeking behavior could be mimicked by A_1_-, but not by A_2A_ receptor antagonists [38], even though A_2A_ antagonists per se tend to produce at least as large increases in locomotion that do A_1_R antagonists [35], [36], [39].

Although a link between the perinatal caffeine use and dopaminergic mechanisms is possible the above data and considerations suggest that the relationship is not simple. Whereas the blockade of A_2A_ receptors has been linked to psychostimulation [32], [33], blockade of A_1_ receptors may instead be related to enhanced glutamatergic transmission [1], [16].Our examination of various neurochemical parameters did not provide any direct evidence for a major disruption of the dopaminergic pathways involved in psychostimulation. We did find an elevation of preproenkephalinA mRNA levels in caudate-putamen. Enkephalin-expressing neurons project to globus pallidus, a brain region that has been demonstrated to be smaller in children with attention-deficit hyperactivity disorder [40]. The potential association between perinatal exposure to caffeine and ADHD in later life needs to be further evaluated. The precise mechanisms underlying the behavioural enhancement in the mouse offspring therefore remains to be explained. It was also not the major focus of this study.

Instead the aim was to use the genetic model to try to elucidate whether the enhancement of cocaine responses in the offspring was due to a primary effect on the mother or the fetus/pup. The approach we used with a genetic study makes it possible to investigate this aspect, which would not be possible using drugs (since it is impossible to administer psychoactive drugs only to the dam and not to the fetus, and vice versa). The results very clearly indicate the first possibility. Thus, if the mother partially lacked A_1_ receptors, the offspring displayed hyperactivity during habituations and responded more strongly to cocaine as adults, regardless of their own genotype. Also, the offspring of wild type mothers showed no behavioral changes, even if they themselves lacked A_1_ receptors (i.e. their fathers lacked A_1_ receptors). The apparent lack of importance of the fetal brain A_1_ receptors is supported by other evidence. Even if adenosine A_1_ receptors are present, albeit sparse, in the embryonic brain [41], these receptors appear to be poorly coupled to G proteins [42]. A maternal effect of caffeine related to adenosine A_1_ receptor signalling was also shown in a recent study where adenylyl cyclase inhibition by an adenosine A_1_ receptor agonist was decreased only in the mother but not the fetal brain [43].

What changes that occur specifically in the pregnant mice that brings about the long-lasting behavioral effects we demonstrate here in the offspring is not known, and will need consideration in future studies. Our experiments performed on the second generation of mice whose grandmother was A_1_R Hz but mother a WT still showed increased response to stimulation with cocaine compared to the WT mice whose both mother and grandmother were wild types. This indicates that long-term behavioral alterations in the offspring may greatly depend on a maternal effect of caffeine and not a direct effect in the fetus, and that some epigenetic effect may be involved.

Epigenetic change to the genome (e.g. in DNA methylation) not only determine the phenotype of the offspring but can sometimes be passed on to the second generation. These processes of transgenerational passage of changes in genomic DNA methylation can occur in both female and male lineage, as the transmission is only via the gamets and can equally apply to sperm and ova [44]. Therefore, epigenetical transmission from father or mother could have the effects on the developmental responses in the offspring. In our study, we have controlled for the mother's and grandmother's genotype in A_1_R Hz offspring but not for the father's or grandfather's genotype. There were two reasons for not considering the paternal genotype. Firstly we wanted to relate our findings to the exposure of mouse dams to caffeine. Clearly paternal effects can not be very important here. Secondly we noted in a separate study that life-long exposure (including perinatal exposure) to caffeine did not cause behavioral changes in the male but did in female mice (Salmi P, Fredholm BB unpublished data).

In summary, we found that adenosine A_1_ receptor heterozygous offspring had a behavioral profile of hyperactivity quite similar to normal mice exposed perinatally to caffeine. Furthermore, it appeared that the genotype of the dam, not the offspring, was critical for behavioral changes in adult offspring. We hypothesize that perinatal caffeine, by acting on adenosine A_1_ receptors in the mother, appears to be responsible for the long-lasting behavioral changes in the offspring. It should be remembered, however, that at this point it is not possible to conclude whether the long-lasting behavioral changes observed here are detrimental or beneficial.

## Materials and Methods

### Animals

Three types of mice were used: *adenosine A_1_ receptor knock-out* (A_1_R KO) [16], *mice heterozygous for the adenosine A_1_ receptor* (A_1_R Hz) and *C57BL/6 mice* (wild type, WT). For generating the A_1_R KO mice, the second coding exon of the mouse adenosine A_1_ receptor gene was inactivated in mouse E14.1 embryonic stem cells. 129/OlaHsd/C57Bl/6 hybrid mice were generated. These animals were backcrossed for at least 10 generations with C57Bl/6 to achieve practically congenic A_1_R KO mice. Animals were bred at the Department of Physiology and Pharmacology, Karolinska Institutet and housed at a constant room temperature (22°C; 12 h light/dark cycle, lights on at 6 a.m) with *ad libitum* access to food and water. To generate the A_1_R KO and A_1_R Hz mice heterozygote matings were usually performed, except where otherwise stated. Mice were genotyped by PCR. All experiments were approved by the Local Committee on Ethics of Animal Experimentation, Stockholm, Sweden.

### Chemicals

Caffeine (anhydrous; Sigma Chemical Co., St. Louis, MO) was administered through the animals' drinking water (0.3 g/l). Cocaine hydrochloride (Apoteksbolaget AB, Sweden) was dissolved in 0.9% NaCl (10 mg/ml) and injected intra-peritoneally (i.p.) in a dose of 10 mg/kg. Saline vehicle served as controls.

### Exposure to caffeine

The experimental design of caffeine exposure is shown in Fig. 1. Pregnant WT females, housed in individual cages, were administered caffeine through their drinking water. The first set of animals received 0.3 g/l caffeine and the second was given tap water only, and served as control. The exposure time ranged from gestational day (GD) 1 to day 21 of lactation, i.e. postnatal day (PND) 21. That is why the term “perinatal” is here used to denote the period of pregnancy and the 21 days after birth. The offspring was analyzed with behavioral tests at two months of age.

#### Behavioral evaluation

All behavioral tests were performed during the light period between 8 a.m. and 4 p.m. The animals were separated into groups based on exposure (caffeine, water, A_1_R Hz from A_1_R Hz mothers) and stimulation (cocaine or saline). Males and females were analyzed separately (n = 6–25 mice in each exposure group, selected randomly from 2–4 independent litters). The motor activity in an open field arena was analyzed at adult age with an experimental protocol that spanned over 4 days (Fig. 1). The rotarod test, including the training session, was performed during the first day. Mice were allowed to rest the next day, and on the third day two open field habituation sessions were performed. The challenge with cocaine, preceded by another habituation session, was done on the fourth day. For this purpose the mice from each exposure group were divided into two sets, one stimulated with cocaine, the other receiving saline. A_1_R KO and their WT littermates were also analysed according to the 4 day protocol explained above.

Regarding adenosine A_1_ receptor heterozygous mice, and their wild type littermates, four different groups of offspring were tested: 1) wild type mice born to an adenosine A_1_ receptor heterozygous mother, 2) adenosine A_1_ receptor heterozygous mice born to an adenosine A_1_ receptor heterozygous mother, 3) adenosine A_1_ receptor heterozygous mice born to a wild-type mother, and 4) wild type mice born to a wild type mother. Behavioral tests were performed when offspring were at least 8 week of age. A_1_R Hz mice were separated into groups described above and challenged with cocaine.

For the evaluation of the effect of knocking out one copy of the A_1_R gene on the second generation of the offspring we have examined the behaviour of the WT male mice whose mothers were wild type but grandmothers were either WT or A_1_R Hz mice. These mice underwent the same behavioural protocol as described in above (Fig. 1).

### Open field model

The effects of caffeine on motor functions were analyzed by recording the locomotor activity in a square open field arena (500×500×225 mm), enclosed in a solid and sound-attenuating box (Kungsbacka Mät och Reglerteknik AB, Fjärås, Sweden). The open field arena was equipped with two rows of photocells sensitive to infrared light, each row having 16 photocells per side. The space between the photocells was 31 mm and the outermost was placed 17.5 mm from the wall. The number of photocell interruptions was collected by a computer and the following variables were recorded and analysed: horizontal activity (Ha, total number of beam breakings), locomotion (L, interruptions of photocells in the lower rows when there is a new beam broken, i.e. the animal has made an actual transfer) and rearing activity (Ra, all interruptions of photocells in the upper rows). This equipment does not allow recording small movements, e.g. tremor, reflexes and tail movements. The data were subjected to a square root transformation (sqrt) before statistical analysis.

Prior to the recording, all animals were allowed a period of 30–45 min in behavioral the testing room. At 2 months of age the mice were analyzed with an experimental protocol that spanned 2 days. Two habituations (45 min each) were performed on day 1, separated by 2 h period. Directly after the first habituation (habituation 3) on the second day the mice were injected with cocaine (10 mg/kg) and their locomotor activity was recorded for 90 min. A_1_ receptor heterozygous mice were separated into groups described in 2.3. and challenged with cocaine.

### Rotarod

Potential effect of the perinatal caffeine exposure on the cerebellum was tested by Rotarod test at 2 months of age (LSi Letica Scientific Instruments, Debiomed, Cornella, Spain). Mice were initially trained to remain on the rotating drum at a constant speed of 4 r.p.m. (revolutions per minute) for 3 min. During the three trials the rotarod accelerated from 4 to 40 r.p.m. over a 5 min period. Two mice were tested simultaneously, and every animal was involved in three consecutive trials, each separated by a 30 min resting period. Fall latencies were recorded. Analysis was performed using the best value obtained in 3 consecutive sessions for each animal.

### Tissue preparation

After the experiments (approximately 2 h after cocaine or vehicle injection) mice were anaesthetized with CO_2_ and sacrificed by decapitation. The brains were dissected out, frozen on dry ice and stored at −80°C. Frozen brains were cut by cryostat into 14 µm coronal sagittal sections, thaw-mounted on poly-L-lysine coated slides as previously described [45], [46] and stored at −20°C.

### In situ hybridization and receptor autoradiography

The immediate early genes NGFI-A and c-fos were examined by in situ hybridisation on coronal sections (14 µm). Expression of preprotachykinin, prodynorphin, prosomatostatin, preproenkephalin and tyrosine hydroxylase mRNA was also measured [39], [47]. Binding studies included dopamine D_1_ receptor binding examined by 0.2, 0.4, 0.6, 1 and 2 nM [^3^H]SCH23390 ((R)-(+)-7-chloro-8-hydroxy-3-methyl-1-phenyl-2,3,4,5-tetrahydro-1H-3-benzazepine, DuPont NEN, Stevenage, UK), D_2_ receptor binding with 1, 2, 3, 5 and 10 nM [^3^H]raclopride (DuPont NEN, Stevenage, UK), respectively as described before [48] and 10 nM [^3^H]mazindol binding [49]. A_1_R binding was evaluated by incubating the brain section from WT and A_1_R Hz animals with increasing (0.2–10 nM) concentrations of A_1_R antagonist [^3^H]1,3-dipropyl-8-cyclopentylxanthine ([^3^H]DPCPX) as described in [50].

Films were developed after 4 weeks of exposure. The autoradiographic films were digitized using a CCD camera (Sierra Scientific Sunnyvale, CA, USA), and optical densities were converted to fmol/mg tissue using a Kodak density wedge and the MCID M5 system (Imaging Research, St. Catharines, Canada). Specific binding was obtained by subtracting non-specific binding from total binding.

### Statistical analysis

Data were analyzed using GraphPad Prism 4 (GraphPad Software Inc., San Diego, USA). To determine statistical significance, open field data were first normalized with a square root transformation. Accumulated counts (horizontal activity and rearing) and rotarod data (fall latencies) were analysed using Student's t test. In some cases Two Way ANOVA was used, as indicated. Differences were considered statistically significant at p≤0.05. Data are presented as means±S.E.M.

## Supporting Information

Figure S1 Rotarod measurements.Analysis of the rotarod performance in adult WT and A_1_R Hz male (A) and female (B) mice that received perinatally 0.3 g/l caffeine or tap water. Fall latencies (mean±S.E.M.) were analyzed by Student's t test (males WT H_2_O: n = 21, WT Caff: n = 7, A_1_R Hz: n = 8; females WT H_2_O: n = 22, WT Caff: n = 13, A_1_R Hz: n = 8).
(3.52 MB TIF)
